# A framework for assessing Health Economic Evaluation (HEE) quality appraisal instruments

**DOI:** 10.1186/1472-6963-12-253

**Published:** 2012-08-16

**Authors:** Astrid Langer

**Affiliations:** 1 Institute of Health Economics and Health Care Management, Munich School of Management, Ludwig-Maximilians Universität München, Munich, Germany; 2 Institute of Health Economics and Health Care Management, Helmholtz Zentrum München – German Research Centre for Environmental Health, Neuherberg, Germany

## Abstract

**Background:**

Health economic evaluations support the health care decision-making process by providing information on costs and consequences of health interventions. The quality of such studies is assessed by health economic evaluation (HEE) quality appraisal instruments. At present, there is no instrument for measuring and improving the quality of such HEE quality appraisal instruments. Therefore, the objectives of this study are to establish a framework for assessing the quality of HEE quality appraisal instruments to support and improve their quality, and to apply this framework to those HEE quality appraisal instruments which have been subject to more scrutiny than others, in order to test the framework and to demonstrate the shortcomings of existing HEE quality appraisal instruments.

**Methods:**

To develop the quality assessment framework for HEE quality appraisal instruments, the experiences of using appraisal tools for clinical guidelines are used. Based on a deductive iterative process, clinical guideline appraisal instruments identified through literature search are reviewed, consolidated, and adapted to produce the final quality assessment framework for HEE quality appraisal instruments.

**Results:**

The final quality assessment framework for HEE quality appraisal instruments consists of 36 items organized within 7 dimensions, each of which captures a specific domain of quality. Applying the quality assessment framework to four existing HEE quality appraisal instruments, it is found that these four quality appraisal instruments are of variable quality.

**Conclusions:**

The framework described in this study should be regarded as a starting point for appraising the quality of HEE quality appraisal instruments. This framework can be used by HEE quality appraisal instrument producers to support and improve the quality and acceptance of existing and future HEE quality appraisal instruments. By applying this framework, users of HEE quality appraisal instruments can become aware of methodological deficiencies inherent in existing HEE quality appraisal instruments. These shortcomings of existing HEE quality appraisal instruments are illustrated by the pilot test.

## Background

The optimal allocation of scarce resources for the production of health benefits to society requires best evidence of cost-effectiveness, and is relevant to any decision in health care. Health economic evaluations support the health care decision-making process by providing information on costs and consequences of health interventions. For example, the NHS Economic Evaluation Database currently contains over 11,000 quality assessed economic evaluations, the results of which are increasingly used for pricing and reimbursement decisions.

To be useful, health economic evaluation studies should be methodologically comparable, of high quality (e.g., in terms of transparency, quality of data sources, completeness of documentation), and relevant for the health care decision context. However, the quality of the conduct and reporting in such studies varies [1]. Health economic evaluation studies are heterogeneous with respect to purposes, conceptual and measurement issues, and value judgments leading to problems with comparability and suboptimal delivery of care. To ensure the scientific quality of such studies, and to facilitate the comparison and transferability of economic evaluation results, methodological standards for health economic evaluations have been established [2]. Besides the purposes of setting methodological and ethical standards, such standards are also used as a formal requirement prior to reimbursement [3].

There are different instruments to guide the quality assessment of health economic evaluations. Among these health economic evaluation (HEE) quality appraisal instruments, considerable agreement exists on the terminology of economic evaluation, the importance of considering alternatives, the need for specifying the analytical viewpoint, the superiority of incremental analysis, the principal need for discounting costs and benefits, and the importance of conducting a sensitivity analysis [2]. Besides particular methodological issues such as inclusion of indirect costs or choice of discount rate, differences between these HEE quality appraisal instruments refer to the level of aggregation. For instance, the International Society for Pharmacoeconomics and Outcomes Research (ISPOR) has set up several task forces for specific elements of an economic evaluation, e.g., the ISPOR Task Force on Good Research Practices for Modeling Studies [4].

As methods of health economic evaluation mature over time, it is also important to appraise and monitor the quality of these HEE quality appraisal instruments which are used by researchers, journals, institutions and governments throughout the world to assess new health technologies and allocate resources. To date, tools for comparing, measuring, and improving the quality of HEE quality appraisal instruments have not been developed. Therefore, the objectives of this study are to: establish a framework for assessing the quality of HEE quality appraisal instruments in order to support and improve their quality; apply this quality assessment framework to those HEE quality appraisal instruments which have been subject to relatively more scrutiny than others, in order to test the framework and to demonstrate the shortcomings of existing HEE quality appraisal instruments.

## Methods

Before developing a framework for quality assessment of HEE quality appraisal instruments, it might be prudent to review experiences regarding other applications of quality appraisal instruments. The obvious locus for such a review is appraisal tools for clinical guidelines.

To identify potentially relevant clinical guideline appraisal instruments, the following electronic databases were searched from inception to October 2010: PubMed, RePEc, and Web of Science. The literature search used the following search terms (including synonyms and closely related words): “clinical guidelines” combined with “appraisal”, “instrument”, and “quality”. Only publications in English, French, or German were considered. Further publications were retrieved by citation tracking, using the “related citations” function in PubMed and Web of Science, hand searching the journals “International Journal of Technology Assessment in Health Care”, “International Journal for Quality in Health Care”, and “Quality & Safety in Health Care” from 2000 to 2010, and searching relevant websites. A total of 14 relevant guideline appraisal instruments were retrieved by the search process (see Additional file 1), which are described in Table 1.

**Table 1:** Critical appraisal tools for clinical practice guideline evaluation

**Author, year, country**	**Journal**	**Title**	**Purpose**	**Items**	**Quality domains**
Brouwers et al. [5], 2010, Canada/ Europe	Journal of Clinical Epidemiology	AGREE II: Advancing guideline development, reporting and evaluation in health care	To assess the process of guideline development	23	6 (scope and purpose; stakeholder involvement; rigor of development; clarity of presentation; applicability; editorial independence)
Cluzeau et al. [6], 1999, UK	International Journal for Quality in Health Care	Development and application of a generic methodology to assess the quality of clinical guidelines	To assess the quality of clinical guidelines	37	3 (rigor of development; clarity of presentation; implementation issues)
Grilli et al. [7], 2000, Italy	The Lancet	Practice guidelines developed by specialty societies: the need for a critical appraisal	To review the quality of guidelines	3	None specified
Hayward et al. [8], 1993, Canada/USA	Annals of Internal Medicine	More informative abstracts of articles describing clinical practice guidelines	To assess the applicability, importance, and validity of guidelines	9	None specified
Helou et al. [9], 1998, Germany	Zeitschrift für Ärztliche Fortbildung und Qualitätssicherung	Methodological quality of clinical practice guidelines in Germany: results of a systemic assessment of guidelines presented on the Internet	To assess the methodological quality of clinical practice guidelines	41	3 (rigor of development; content and format; applicability)
Institute of Medicine [10], 1992, USA	-	Guidelines for clinical practice: from development to use	To examine the soundness of guidelines and to encourage their systematic development	46	7 (validity; clarity; multidisciplinary process; clinical flexibility; reliability and reproducibility; clinical adaptability; scheduled review)
Liddl et al. [11], 1996, Australia	-	Method for evaluating research guideline evidence	To assess the validity of existing guidelines or to summarize the validity of guidelines during their development	14	3 (descriptive information about the guideline; evaluation criteria for the guideline; overall assessment of the guideline)
Marshall [12], 2000, Canada	The Canadian Journal of Gastroenterology	A critical approach to clinical practice guidelines	To evaluate the quality, relevance and effectiveness of clinical practice guidelines	9	None specified
Mendelson [13], 1995, USA	Radiologic Clinics of North America	The development and meaning of appropriateness guidelines	To establish appropriateness criteria for guidelines	8	None specified
Scottish Intercollegiate Guidelines Network [14], 2008, Scotland	-	SIGN 50: A guideline developer’s handbook	To assess the process of guideline development	23	6 (see Brouwers et al.)
Selker [15], 1993, USA	The American Journal of Cardiology	Criteria for adoption in practice of medical practice guidelines	To establish criteria for adoption of practice guidelines for clinical practice	8	None specified
Shaneyfelt et al. [16], 1999, USA	Journal of the American Medical Association	Are guidelines following guidelines? The methodological quality of clinical practice guidelines in the peer-reviewed medical literature	To review the methodological quality of clinical practice guidelines in the peer-reviewed medical literature	25	3 (guideline development and format; evidence identification and summary; formulation of recommendations)
Shiffman et al. [17], 2003, USA	Annals of Internal Medicine	Standardized reporting of clinical practice guidelines: a proposal from the Conference on Guideline Standardization	To establish a standard for guideline reporting	18	None specified
Ward, Grieco [18], 1996, Australia	Medical Journal of Australia	Why we need guidelines for guidelines: a study of the quality of clinical practice guidelines in Australia	To assess the quality of clinical practice guidelines in Australia	18	8 (validity; reproducibility; applicability; clinical flexibility; clarity; multidisciplinary process; documentation; scheduled review)

However, it was not intended to provide a systematic review of appraisal tools for clinical practice guideline evaluation. Therefore, the interested reader is referred to the reviews by Vlayen et al. [19] and Graham et al. [20], which were identified by the literature search on clinical guideline appraisal instruments and provide a detailed description and comparison of clinical practice guideline appraisal instruments. These systematic reviews were used to inform the framework development.

Based on a deductive iterative process, the clinical guideline appraisal instruments identified were reviewed, consolidated, and adapted. For this purpose, all questions/statements included in these instruments were listed to exclude double counting. For inclusion in the final framework, the questions/statements were required to have the following characteristics:

○ Generally and internationally accepted

○ Relevant to the realm of health economic evaluation

○ Distinguishable from other questions/statements (i.e., the questions/statements selected for final inclusion should overlap as little as possible)

The questions/statements identified by this method were thematically grouped to devise the final quality assessment framework for HEE quality appraisal instruments.

In order to test the framework and demonstrate the shortcomings of existing HEE quality appraisal instruments, the framework developed was applied to those HEE quality appraisal instruments which have been subject to relatively more scrutiny than others. Using a similar search process, HEE quality appraisal instruments were determined possibly relevant if they provided explicit criteria against which the quality of economic evaluations could be appraised. Because of regional, cultural, institutional, or political preferences and interests, country-specific guidelines were not considered for inclusion. Instruments assessing the transferability of health economic evaluations were also beyond the scope of this study.

## Results

### Framework for quality assessment of HEE quality appraisal instruments

The framework consists of 36 items organized within 7 dimensions, each of which captures a specific domain of quality: Dimension A (“*purpose and scope*”, items A1-A5), Dimension B (“*stakeholder involvement*”, items B1-B3), Dimension C (“*rigor of development process/validity*”, items C1-C8c), Dimension D (“*reliability/ reproducibility*”, items D1-D4d), Dimension E (“*clarity of presentation*”, items E1-4), Dimension F (“*applicability*”, items F1-F5), *Dimension G (“evaluation*”, items G1-G2). The quality assessment framework is presented in Table 2.

**Table 2:** Framework for quality assessment of HEE quality appraisal instruments

**Dimension**	**Item**
*A. Purpose and scope*	A1. The reasons for developing the guideline are stated
	A2. The overall objective of the guideline is described
	A3. The health economic studies for which the guideline was designed are stated
	A4. The target audience of the guideline is characterized
	A5. The time frame to which the guideline is meant to apply is specified
*B. Stakeholder involvement*	B1. The guideline development group consists of individuals from all relevant disciplines
	B2. Conflicts of interest of guideline development group members have been recorded and addressed
	B3. The views and preferences of the target audience have been sought
*C. Rigor of development process/validity*	C1. The members of the guideline development group and their affiliations are stated
	C2. The methods used for literature search are specified
	C3. The sources of evidence on which the guideline is based are described
	C4. The criteria for selecting existing evidence are described
	C5. The methods for formulating the items are specified
	C6. The methods used to reach consensus are specified
	C7. The date for reviewing/updating the guideline is stated
	C8. The guideline is valid in terms of:
	*C8a. Content (internal) validity*
	*C8b. Criterion (external) validity*
	*C8c. Construct validity (convergent and discriminant validity)*
*D. Reliability/ reproducibility*	D1. The process of guideline development is documented
	D2. The guideline has been externally reviewed by experts prior to its publication
	D3. The guideline has been piloted/pretested among the target audience
	D4. The guideline is reliable in terms of:
	*D4a. Inter-rater reliability*
	*D4b. Test-retest reliability*
	*D4c. Parallel-forms reliability*
	*D4d. Internal consistency reliability*
*E. Clarity of presentation*	E1. The items of the guideline are specific and clearly worded
	E2. The items of the guideline are clearly presented and user friendly
	E3. The guideline can be used in a straightforward manner
	E4. Key items are easily identifiable
*F. Applicability*	F1. The guideline provides a standard reporting format
	F2. The guideline gives a detailed assessment instruction
	F3. The guideline presents items of methodological quality and transparency
	F4. The guideline uses a quality score
	F5. The strengths and limitations of the guideline are specified
*G. Evaluation*	G1. The methods for evaluating the guideline are described
	G2. The adherence to the guideline by the target audience is described

### Application of the quality assessment framework

To test the established framework and to demonstrate the shortcomings of existing HEE quality appraisal instruments, four well-known, often-cited, and widely-used HEE quality appraisal instruments, which have been subject to relatively more scrutiny than most others, were selected: the Quality of Health Economic Studies (QHES) instrument developed by Chiou et al. [21], the British Medical Journal (BMJ) guidelines for economic submissions established by Drummond and Jefferson on behalf of the BMJ Economic Evaluation Working Party [22], the Consensus on Health Economic Criteria (CHEC) list devised by Evers et al. [23], and the Good Practice Guidelines for Decision-Analytic Modeling accomplished by Philips et al. [24].

The BMJ list, the CHEC list, and the Philips list were chosen because they are recommended by the Cochrane Handbook for Systematic Reviews of Interventions [25] for critical appraisal of the methodological quality of health economic evaluation studies. In the chapter related to systematic reviews of economic evaluations, the Centre for Reviews and Dissemination’s guidance for undertaking reviews in health care [26] also refers to the BMJ list and the Philips list as instruments to assess the quality of economic evaluations. The QHES instrument was selected because it is an example of a quality scoring system which has been the subject of controversy in the literature.

As can be seen in Table 3, the BMJ list and the QHES instrument are broader and more comprehensive than the other two quality appraisal instruments: this is because the Philips list is solely designed for model-based economic evaluations and the CHEC list is only intended for undertaking systematic reviews of trial-based economic evaluations. In Table 4, the main characteristics of the HEE quality appraisal instruments are provided.

**Table 3:** Scope of the four selected HEE quality appraisal instruments

	**Quality appraisal instrument for economic evaluation**	**Quality appraisal instrument for systematic reviews**
Trial-based economic evaluation	1,2	1,2,3
Model-based economic evaluation	1,2,4	1,2,4

1 QHES instrument [21].

2 BMJ guidelines [22].

3 CHEC list [23].

4 Philips guidelines [24].

**Table 4:** Main characteristics of the four selected HEE quality appraisal instruments

	**QHES instrument**[21]	**BMJ guidelines**[22]	**CHEC list**[23]	**Philips guidelines**[24]
*Author; year; journal*	Chiou et al. 2003; Medical Care	Drummond, Jefferson; 1996; British Medical Journal (BMJ)	Evers et al. 2005; International Journal of Technology Assessment in Health Care	Philips et al. 2006; Pharmacoeconomics
*Affiliation of authors*	Academia and industry	Academia	Academia	Academia
*Published in a peer-reviewed journal*	Yes	Yes	Yes	Yes
*Number of references*	35	48	30	22
*Purpose*	To provide a grading system for assessing the quality of health economic evaluations	To improve the transparency of reporting	To develop a generally accepted criteria list for assessing the methodology of economic evaluation studies in systematic reviews	To identify, review, and consolidate currently available guidelines in order to establish a synthesized and consistent quality assessment framework for decision analytic models
*Development process*	Selection of criteria from 19 existing guidelines; Use of a conjoint analysis survey of 120 international experts to estimate weights for each criterion	Not specified	Selection of items from 15 existing guidelines; Use of a Delphi panel consisting of 23 international experts to generate the final criteria list	Selection and formulation of items by reviewing and consolidating 15 existing guidelines for good practice in decision-analytic modeling in HTA
*Time frame*	Before, during and after peer review	Before, during, and after peer review	After peer review	Before, during, and after peer review
*Target audience*	Producers and consumers of economic evaluations	Producers and consumers of economic evaluations	Consumers intending to conduct a systematic review of trial-based economic evaluations	Producers and consumers of model-based economic evaluations
*Preferred analytical technique*	Full economic evaluations: cost-minimization-, cost-effectiveness-, cost-utility-, cost-benefit-analysis	Full economic evaluations: cost-minimization-, cost-effectiveness-, cost-utility-, cost-benefit-analysis	Full economic evaluations based on clinical trials: cohort studies, case–control studies, randomized controlled clinical trials	Full economic evaluations based on decision-analytic models
*Standard reporting format included*	16 questions which should be asked when appraising the quality of health economic evaluations	Ten sections under the three headings of study design, data collection, and analysis and interpretation of results: study question, selection of alternatives, form of evaluation, effectiveness data, benefit measurement and valuation, costing, modeling, adjustments for timing of costs and benefits, allowance for uncertainty, and presentation of results	19 questions which should be asked when appraising the quality of health economic evaluations in systematic reviews	15 sections under the three key themes of structure, data, and consistency: statement of decision problem/objective, statement of scope/ perspective, rationale for structure, structural assumptions, strategies/comparators, model type, time horizon, disease states/pathways, cycle length, data identification, pre-model data analysis, data incorporation, assessment of uncertainty, internal consistency, and external consistency
*Number of questions/criteria*	16	35	19	61
*Operationalization of the questions/criteria*	Yes/No	Yes/No/Not clear/Not appropriate	Yes/No	Yes/No/Unclear/Not applicable
*Use of a quality score*	Yes	No	No	No
*Assessment instruction*	No	Yes	Yes: http://www.beoz.unimaas.nl/chec/	Yes
*Pilot test of the guideline*	Yes: Ofman et al. [31]	Not specified	Yes, but no details given	Yes

### Pilot review of the quality assessment framework

The experiences of applying the quality assessment framework to the HEE quality appraisal instruments are presented in Table 5.

**Table 5:** Application of the quality assessment framework to the four selected HEE quality appraisal instruments

**Dimension**	**Item**	**QHES instrument**[21]	**BMJ guidelines**[22]	**CHEC list**[23]	**Philips guidelines**[24]
*A. Purpose and scope*	A1	YES	YES	YES	YES
	A2	YES	YES	YES	YES
	A3	YES	YES	YES	YES
	A4	YES	YES	YES	YES
	A5	YES	YES	YES	YES
*B. Stakeholder involvement*	B1	NO	NO	NO	NO
	B2	YES	YES	NO	YES
	B3	YES	YES	YES	YES
*C. Rigor of development process/validity*	C1	YES	PARTIALLY	YES	YES
	C2	YES	NO	YES	YES
	C3	YES	NO	YES	YES
	C4	YES	NO	NO	YES
	C5	YES	NO	YES	YES
	C6	YES	NO	YES	YES
	C7	NO	NO	NO	NO
	C8				
	*C8a*	NO	NO	NO	NO
	*C8b*	NO	NO	NO	NO
	*C8c*	YES	NO	NO	NO
*D. Reliability/reproducibility*	D1	YES	NO	YES	YES
	D2	NO	YES	NO	YES
	D3	YES	NO	NO	NO
	D4				
	*D4a*	NO	NO	NO	NO
	*D4b*	NO	NO	NO	NO
	*D4c*	NO	NO	NO	NO
	*D4d*	NO	NO	NO	NO
*E. Clarity of presentation*	E1	NO	YES	YES	NO
	E2	YES	YES	YES	NO
	E3	NO	NO	NO	NO
	E4	YES	NO	NO	NO
*F. Applicability*	F1	YES	YES	YES	YES
	F2	NO	YES	YES	YES
	F3	YES	YES	YES	YES
	F4	YES	NO	NO	NO
	F5	YES	NO	YES	YES
*G. Evaluation*	G1	NO	YES	YES	YES
	G2	NO	NO	NO	NO

The quality dimension of “*purpose and scope*” (dimension A) is fulfilled by all HEE quality appraisal instruments, even though not all items are explicitly described. In terms of the quality dimension of “*stakeholder involvement*” (dimension B), only Evers et al. (CHEC list, [23]) do not declare whether they have any conflicts of interest. Furthermore, at none of the different HEE quality appraisal instrument development stages were all the key professionals (e.g., economists, clinicians, epidemiologists, and statisticians) involved. However, all the quality appraisal instruments used methods to ensure that the perspectives of the target audience informed the development process (e.g., by participation on the development group, or by external review of drafts of the appraisal instruments). The main differences in the quality of the four HEE quality appraisal instruments relate to the quality dimension of “*rigor of development process/validity*” (dimension C). Especially with the BMJ list, the different stages of the development process are not reported. Moreover, as methodology advances, the date for updating the appraisal instrument should be stated. Only Philips et al. [24] refer to the need for periodic updates, but when these should take place remains unclear. The lack of formal validity is one of the main limitations of all four quality appraisal instruments. Only the QHES instrument was formally validated in terms of construct validity [21]. Other limitations refer to the quality dimension of “*reliability/reproducibility*” (dimension D). As mentioned before, the development process of the BMJ list is not documented. All four HEE quality appraisal instruments were published in peer-reviewed journals and, hence, external reviewers were involved in appraisal instrument development during the generic review process. However, in order to develop the BMJ list and the Philips list, additional external experts were convened to discuss drafts of these quality appraisal instruments. In addition, provided that external reviewers should not have been involved in developing the appraisal instrument, the QHES instrument and the CHEC list have not been externally reviewed before their publication (certainly, except for the review process). For the appraisal instrument to be effective with regard to reliability and reproducibility, it also needs to be piloted/pretested among the target audience. Only the QHES instrument was pretested among 60 experts in the field of health economics, who evaluated the methodological quality of three health economic analyses, first on a visual analog scale, and then using the grading system. In respect of the quality dimension of “*clarity of presentation*” (dimension E), the appraisal instruments established by Philips et al. [24] and the grading system developed by Chiou et al. (QHES instrument, [21]) do not provide specific and unambiguous items in those cases where more than one question refers to the same criterion, resulting in ambivalent assessments. Additionally, on account of the rather technical nature of the questions provided by Philips et al. [24], these are only suitable for specialist readers with expertise in the field of decision-analytic modelling and with knowledge of the disease area. Philips et al. [24] state that without that knowledge, it is a complex matter to decide whether all structural assumptions are justified, or whether all feasible and practical options have been evaluated. Further, because of the problem with the interpretation of the term “justified” or “appropriate”, it might be difficult to use these four quality appraisal instruments in a straightforward manner. Some items/questions are highly dependent on the judgment of the respective user and thus have an unavoidable element of subjectivity. For example, based on a comparison of three instruments for appraising the quality of health economic evaluations (BMJ list, CHEC list, and QHES instrument) Gerkens et al. [27] found that the reviewer has a greater influence on the results of the quality assessment than the appraisal instrument itself. Another problem within this dimension concerns the operationalization of the questions. Chiou et al. (QHES instrument, [21]) and Evers et al. (CHEC list, [23]) provide questions in a yes/no format, but in specific circumstances some questions may not be applicable to the study context. Because of the weighting of the criteria, only the key items of the QHES instrument can be easily identified. Regarding the quality dimension of “*applicability*” (dimension F), all appraisal instruments provide a standard reporting format and present items of methodological quality and transparency. All but the QHES instrument give detailed assessment instructions (to a greater or lesser extent) and all appraisal instruments except for the BMJ list specify their strengths and limitations. The QHES instrument is the only quality appraisal instrument to use a quality score. Concerning the quality dimension of “*evaluation*” (dimension G), all but the QHES instrument describe the evaluation methods. However, none of the instruments describes the extent of adherence by the target audience.

## Discussion

At present, there is no common instrument for measuring and improving the quality of HEE quality appraisal instruments. A quality assessment framework for HEE quality appraisal instruments was developed to support and improve their quality. It permits not only the assessment of their quality but also the recognition of the most urgent adjustments needed to improve their quality. Applying the quality assessment framework to four existing HEE quality appraisal instruments, it was found that these quality appraisal instruments are of variable quality.

Moreover, the HEE quality appraisal instruments have other limitations. The CHEC list established by Evers et al. [23] consists of a minimum set of items and is intended only for full economic evaluations based on clinical trials. In order to appraise the overall methodological quality of trial-based economic evaluations, the authors point out that their list should be used in combination with existing instruments for assessing the quality of clinical trials [23]. In systematic reviews including trial-based and model-based economic evaluations, the CHEC list should be combined with issues relevant to modelling studies such as structural assumptions. Therefore, in a systematic review [28], the appraisal instruments developed by Philips et al. [29] were used as a complement to the CHEC list. Another limitation concerns the items included in the CHEC list. As these items should deliver insight into the quality of economic evaluation studies, most of them are rather subjective, which is a challenge for the inter-rater reliability [23]. However, formularies and HTAs would in fact need some flexibility to make their own best decisions for their patients. The subjective judgment generally inherent in quality assessment is a particularly fundamental problem for the Philips guidelines [24]. Additionally, because of the combination of transparency and quality aspects in the same question, the Philips guidelines produce ambivalent quality assessments. By contrast, other research groups provide quality appraisal instruments that differentiate between these two issues [30]. Furthermore, because of the scope of the Philips list, it should be used in conjunction with more general quality assessment instruments for health economic evaluation (e.g., the BMJ list) [24]. The quality appraisal instrument devised by Philips et al. [24] include dimensions of methodological quality corresponding to rationales for structure, structural assumptions, disease states/pathways, cycle length, and internal consistency. This is due to the fact that this HEE quality appraisal instrument is specific for decision-analytic models, and, thus, has a more technical character than the other three HEE quality appraisal instruments. Therefore, the Philips list does not highlight the importance of discounting, the superiority of incremental analysis, and the measurement and valuation of costs. However, in their version published in 2004, Philips et al. [29] point out that “costing and discounting methods should accord with standard guidelines for economic evaluation”. Due to limitations in reporting, the quality of the BMJ list in particular was difficult to assess. In contrast to the other three quality appraisal instruments, the QHES instrument provides a grading system, but the advantage of scoring methods is questionable.

To date, relatively little empirical research has been undertaken in order to investigate the influence of decisions to include only economic evaluations of high quality on the results of a critical assessment of health economic evaluations. However, such lessons can be obtained from the experiences made with quality scores for clinical studies. Using 25 different quality assessment scales to identify high-quality clinical trials, Jüni et al. [32] show that the conclusions of meta-analytic studies of randomized clinical trials might be affected by the type of quality assessment scale used. They consider the use of grading systems to be problematic, and thus they recommend that relevant methodological issues should be appraised individually. In a review, Moher et al. [33] use the same 25 scales to show differences in scale development. As these differences can result in important differences in quality assessment, they recommend that meta-analyses of randomized clinical trials should be undertaken with and without assessing quality. Based on these experiences, it is not recommended to select economic evaluations on the basis of their quality scores, as proposed by Chiou et al. (QHES instrument, [21]. In general, a corresponding NHS EED structured abstract [34] consisting of “subject of study”, “key elements of study”, “details about clinical evidence”, “economic analysis”, “results”, “critical commentary”, “implications”, and “other publications of related interest” might enhance quality assessment of all types of full health economic evaluation informed by HEE quality appraisal instruments, because these abstracts provide critical appraisal of methodological quality on the basis of the same quality dimensions as included in the quality appraisal instruments [25]. In their study, Thurston et al. [35] found that decision-makers in health care need an initial screen of economic evaluation results provided by a critical descriptive summary or a score, plus a short abstract to gather more information on the quality and relevance of economic evaluation results. However, how to condense information provided by critical appraisal of methodological quality is an unresolved issue which requires further research.

## Conclusions

The framework described in this study should be regarded as a starting point for assessing the quality of HEE quality appraisal instruments. This framework can be used by quality appraisal instrument producers to support and improve the quality and acceptance of existing and future HEE quality appraisal instruments. By applying this framework, users of HEE quality appraisal instruments can become aware of methodological deficiencies inherent in existing quality appraisal instruments, as illustrated by the pilot test. As the development of HEE quality appraisal instruments is a dynamic and interactive process, and as methodology advances, a continual update of existing quality appraisal instruments is needed.

## Competing interests

The author declares that she has no competing interests.

## Pre-publication history

The pre-publication history for this paper can be accessed here:

http://www.biomedcentral.com/1472-6963/12/253/prepub

## Supplementary Material

Additional file 1 **Flow chart for study selection for clinical practice guideline evaluation.** Flow chart showing the records identified through database searching and other sources during the literature search for clinical practice guideline evaluation. Click here for file
