# Supplementary material for: A framework for assessing Health Economic Evaluation (HEE) quality appraisal instruments
Source: BMC Health Serv Res. 2012 Aug 16;12:253. doi: 10.1186/1472-6963-12-253 (PMC3507835; doi:10.1186/1472-6963-12-253)
Supplement: Additional file 1 — Flow chart for study selection for clinical practice guideline evaluation. Flow chart showing the records identified through database searching and other sources during the literature search for clinical practice guideline evaluation. [file 1472-6963-12-253-S1.doc]

## Additional file 1 - Flow chart for study selection for clinical practice guideline evaluation

Records identified through database searching

(n = 159)

Additional records identified through other sources

(n = 116)

Records after duplicates removed

(n = 141)

Records screened

(n = 141)

Records excluded*

(n = 3)

Full-text articles assessed for eligibility

(n = 138)

Full-text articles excluded**

(n = 124)

Studies included in qualitative synthesis

(n = 14)

*Published in other languages than English, French or German

**Wrong publication type (letter, editorial, review, other); no instrument for guideline evaluation
